# Corrigendum to “Nurses' Recovery Experiences during the COVID-19 Pandemic in Isfahan, Iran: A Qualitative Study”

**DOI:** 10.1155/2023/9825318

**Published:** 2023-12-22

**Authors:** Azam Hosseinzadeh, Akbar Etebarian Khorasgani, Alborz Gheitani, Reza Ebrahimzadeh, Roya Torkashvand

**Affiliations:** ^1^Department of Public and Media Management, Isfahan (Khorasgan) Branch, Islamic Azad University, Isfahan, Iran; ^2^Department of Nursing, Isfahan (Khorasgan) Branch, Islamic Azad University, Isfahan, Iran

In the article titled “Nurses' Recovery Experiences during the COVID-19 Pandemic in Isfahan, Iran: A Qualitative Study” [1], there was an error in affiliations 1 and 2. In the corrected affiliations, the branch has been revised from “University of Isfahan (Khorasgan) Branch” to “Isfahan (Khorasgan) Branch.” The corrected affiliation appears below:

1. Department of Public and Media Management, Isfahan (Khorasgan) Branch, Islamic Azad University, Isfahan, Iran
2. Department of Nursing, Isfahan (Khorasgan) Branch, Islamic Azad University, Isfahan, Iran
